# Joint modelling of malaria and anaemia in children less than five years of age in Malawi

**DOI:** 10.1016/j.heliyon.2021.e06899

**Published:** 2021-05-04

**Authors:** Rugiranka Tony Gaston, Shaun Ramroop, Faustin Habyarimana

**Affiliations:** a School of Mathematics, Statistics and Computer Sciences, University of KwaZulu-Natal, Pietermaritzburg Campus, Private Bag X01, Scottsville, 3209, South Africa; b Health Economics and HIV/AIDS Research Division (HEARD), University of KwaZulu-Natal, Westville Campus, Private Bag X01, Westville, 3629, South Africa

**Keywords:** Malaria, Anaemia, Correlation, Children under five years of age, Multivariate joint model, MMIS 2017, Public health, Infection disease, Nutrition

## Abstract

**Background:**

Malaria and anaemia jointly remain a public health problem in developing countries of which Malawi is one. Although there is an improvement along with intervention strategies in fighting against malaria and anaemia in Malawi, the two diseases remain significant problems, especially in children 6–59 months of age. The main objective of this study was to examine the association between malaria and anaemia. Moreover, the study investigated whether socio-economic, geographic, and demographic factors had a significant impact on malaria and anaemia.

**Data and methodology:**

The present study used a secondary cross-sectional data set from the 2017 Malawi Malaria Indicator Survey (MMIS) with a total number of 2 724 children 6–9 months of age. The study utilized a multivariate joint model within the ambit of the generalized linear mixed model (GLMM) to analyse the data. The two response variables for this study were: the child has either malaria or anaemia.

**Results:**

The prevalence of malaria was 37.2% of the total number of children who were tested using an RDT, while 56.9% were anaemic. The results from the multivariate joint model under GLMM indicated a positive association between anaemia and malaria. Furthermore, the same results showed that mother's education level, child's age, the altitude of the place of residence, place of residence, toilet facility, access to electricity and children who slept under a mosquito bed net the night before the survey had a significant effect on malaria and anaemia.

**Conclusion:**

The study indicated that there is a strong association between anaemia and malaria. This is interpreted to indicate that controlling for malaria can result in a reduction of anaemia. The socio-economic, geographical and demographic variables have a significant effect on improving malaria and anaemia. Thus, improving health care, toilet facilities, access to electricity, especially in rural areas, educating the mothers of children and increasing mosquito bed nets would contribute in the reduction of malaria and anaemia in Malawi.

## 1 Introduction

Malaria and anaemia jointly remain a public health problem worldwide in both developed and developing countries (Kanchana et al., 2018). Malaria and anaemia are life-threatening diseases, mainly in developing countries, where children and pregnant women are more vulnerable (McLean et al., 2009; WHO, 2017; Kejo et al., 2018). The two conditions are known to add to the tremendous weight of morbidity and mortality, especially in children under five years of age (Wanzira et al., 2017; White, 2018). The significant progress in fighting both malaria and anaemia has been improved worldwide. However, both diseases remain a health problem especially in children from developing countries, and Malawi is among the developing countries (WHO, 2015; Kejo et al., 2018; Roberts and Zewotir, 2020).

Malaria is caused by different *Plasmodium* parasites, such as *P. falciparum, P. vivax, P. ovale, P. malariae* and *P. knowlesi, with P. falciparum* the most prevalent parasite in the African region (Perkins et al., 2011; WHO, 2015; Seyoum, 2018). Malaria is transmitted in the human body through the bites of infected *Anopheles* mosquitoes, where the malaria parasites infect the red blood cells and reduce the amount of the blood cells, which can lead to severe anaemia (Noland et al., 2012; Seyoum, 2018). The combination of rainfall, humidity and high temperature in low altitudes creates favourable conditions for breeding and development of malaria vectors. Thus, for this reason the transmission of malaria becomes higher when the climate is wet, hot and more humid (Chirombo et al., 2014; Gaston and Ramroop, 2020).

The World Health Organization (WHO) estimated approximately 219 million cases of malaria in 2017, with 200 million from Africa (World Malaria Report, 2018). In addition, the WHO reported the total number of 43 000 deaths from malaria globally in 2017, and 93% of cases were from the African Region. Children under five years accounted for 61% of deaths globally and 93% were from Africa (World Malaria Report, 2018). In 2013, malaria in Malawi accounted for 20% of children under five years who died in hospital, and the prevalence of malaria is still high (WHO, 2016; NMCP and ICF, 2018; Gaston and Ramroop, 2020).

The World Health Organization defines anaemia as a reduction of hemoglobin in blood cells, which causes the body tissues to not have enough oxygen. In women, men or children, anaemia can be categorized as mild, moderate, and severe. In children 6–59 months of age, when the hemoglobin concentration level is less than 11 g/dl, the child is considered anaemic (Korenromp et al., 2004; WHO, 2015; NMCP and ICF, 2018). However, in the case of malaria related anaemia, the cutoff for the hemoglobin concentration level is below 8 g/dl (NMCP and ICF, 2018).

Globally, approximately 1.62 billion people are affected by anaemia and this accounts for more than 24.8% of the world population, around 43% from developing countries, and 47.4% of these are children (McLean et al., 2009; Kanchana et al., 2018). In Malawi, the prevalence of anaemia in children was 63% and this shows that anaemia remains a health problem and more care is needed in the country (NSO and ICF, 2017). Anaemia is detrimental to a child's health in that it can affect children's physical and mental development which can also affect socio-economics (Abegunde and Stanciole, 2006; Magalhaes and Clements, 2011; WHO, 2011; Gaston et al., 2018).

The main causes of anaemia are nutritional deficiencies and infection diseases such as HIV, intestinal worms, intake of iron, folate, vitamin B12, malaria and other parasitic infections (McCuskee et al., 2014; Gaston et al., 2018). However, in the region of malaria-endemic, the main contributor to anaemia is malaria (Brabin et al., 2004; Crawley, 2004; World Malaria Report, 2018). It is known that malaria is the major contributor to anaemia, and a huge amount of mortality and morbidity is caused with both malaria and anaemia (Björkman, 2002; Carneiro et al., 2006; Wanzira et al., 2017). In the area of high prevalence of malaria, anaemia is held accountable for about half of malaria related deaths (Korenromp et al., 2004; Adebayo et al., 2016; Seyoum, 2018). These two diseases are associated and this means that controlling malaria can reduce anaemia, and controlling anaemia can results in reduction of deaths related to malaria (Korenromp et al., 2004; Noland et al., 2012; Reithinger et al., 2013; Hershey et al., 2017). The study by McLean et al. (2009) revealed that more than half of the reduction in malaria resulted in a reduction of 60% in the risk of having anaemia. This confirms that anaemia and malaria are correlated diseases, which can increase mortality and do more damage in children if no actions are taken timeously (Gaston et al., 2018).

There are very few studies on modelling both childhood anaemia and malaria simultaneously as many studies showed that the young children are more vulnerable to both (Hershey et al., 2017; Kuziga et al., 2017; Yimgang et al., 2021). In addition, the health of a child should be prioritized as they are the posterity of the country (Gaston et al., 2018). Hence, in light of the aforementioned reasons, the current study focused on the modelling of anaemia and malaria in children 6–59 months of age in order to understand the link between the two conditions diseases so that they can be controlled and eliminated. Furthermore, it assists in policy-making and planning of interventions strategies from different donors. In Malawi, many researchers were interested in modelling the prevalence of anaemia and malaria in children separately (Chitunhu and Musenge, 2015; Mathanga et al., 2015; Calis et al., 2016; Kabaghe et al., 2017; Ntenda et al., 2017; Zgambo et al., 2017; Hajison et al., 2018; Nkoka et al., 2019). The separated model has its benefits but cannot address the possible association between the two diseases jointly. The joint model is needed to simultaneously model anaemia and malaria to address the association between the two diseases along with identifying factors associated with the diseases. The multivariate joint model under a GLMM has focal points when compared to separate models, for instance, better control of type I error rates in the various tests. Besides this, the multivariate joint model is better for expansion in the capability of the parameter estimate and the ability to address distinctively multivariate questions. Furthermore, the GLMM includes the random effect in the model in order to model the correlation between two or more observations (Gueorguieva, 2001; Hedeker, 2005; Agresti, 2015; Habyarimana et al., 2016; Gaston and Ramroop, 2020).

Relevant literature reveals that numerous researchers proposed different statistical models to analyze the association between malaria and anaemia in children (Safeukui et al., 2015; Adebayo et al., 2016; Seyoum, 2018).

In Malawi, the study by Kabaghe et al. (2017) used a year repeated cross-sectional survey from a rural area in Malawi to analyse the short change in anaemia and malaria under five years’ children. The study by McGann et al. (2018) also used the 2015 DHS data set to describe the prevalence and distribution of inherited blood disorders among young children in Malawi and explore their associations with malaria and anaemia. The recent study by Roberts and Zewotir (2020) used Geospatial maps to visualize the relationship between malaria and anaemia in Malawi, Uganda, Tanzania and Kenya. In addition, the study by Yimgang et al. (2021) evaluated the population attributable fraction of anaemia due to malaria in children between 5-15 years in Southern Malawi. However, according to our knowledge, no study in literature utilized the generalized linear mixed model (GLMM) to simultaneously join malaria and anaemia in children 6–59 months of age in Malawi and this highlights the novelty of the current research. In addition, the data set used is different in comparison to the 2017 Malawi Malaria Indicator Survey (MMIS) data set.

Therefore, the current study aimed to simultaneously model the association between malaria and anaemia and identify factors associated with the two diseases by utilizing the joint model for a multivariate generalized linear mixed model (GLMM) using the 2017 MMIS.

## 2 Methodology and material

### 2.1 Study area

Malawi is one of the African countries and is among the Sub-Saharan African nations located south of the equator and surrounded by Tanzania in the North and Northeast; by Mozambique toward the East and Southwest; and Zambia toward the West and Northwest (NMCP and ICF, 2018). Malawi is divided into three regions and twenty-eight districts; with the Northern region split into six districts, whilst the Central region consists of nine districts, and the Southern region has thirteen districts.

The country has a tropical climate that is conducive for the breeding of *Anopheles* mosquitos and the mosquitos increase in the rainy season from November to April. The climate is cool and dry from May to August during which the transmission of malaria reduces compared to the rainy season (Kazembe, 2007; Zgambo et al., 2017). The economy of the country is based on agriculture and is among the poorest countries in the world with poor healthcare in comparison with other African countries (WHO, 2016).

### 2.2 Data sources

The study utilized secondary cross-sectional data set from the 2017 Malawi Malaria Indicator Survey (MMIS). The data was gathered between 15 April and June 2017 and executed by the Malawi National Malaria Control Program (NMCP) through help from the President's Malaria Initiative (PMI). The United States Agency for International Development (USAID) offered money related help through the President's Malaria Initiative (PMI). They likewise supported the undertaking by offering specialized help with the administration of community and wellbeing studies as they do in nations around the world (NMCP and ICF, 2018). The overseeing group of Malawi gave staff office space, and key assistance. From there on, the ICF offered specialized help through the Demographic and Health Survey (DHS) program. The ethical approval was evaluated and granted by the Malawian Ministry of Health Research and Ethics Committee with the support of the Institutional Review Board of ICF International.

### 2.3 Data sampling and design

Women between 15-49 years of age and children from 6-59 months who stayed in or visited the selected households the night before the survey were included in the interview. The 2017 MMIS was a population based on a household cluster survey and the sampling survey followed the two-stage sample design. Furthermore, the 2017 survey enables assessments of key malaria indicators for the nation as a whole, for urban and rural areas independently, and for all of the three administrative territories in Malawi: Northern, Central, and Southern.

The first stage of sampling comprised a selection of 150 clusters from the enumeration areas (EAs) outlined in the 2008 Population and Housing Census. Among the 150 clusters, 60 were from urban and 90 from rural areas. The second stage of sampling involved the systematic selection of a sample of 3 750 households. Of these households, 25 households were selected from each enumeration area (EA) (NMCP and ICF, 2018).

The study used a total weighted number of 2 724 children 6–59 months of age to establish a national-level portrait (NMCP and ICF, 2018). The study used the weighted sample to gain insights that were illustrative of the nation and to account for the complex sample design from the data set. In the sampling procedure, the individuals surveyed in each region should contribute proportionally to the size of the total sample in the region. In any case, some regions may have small populations, and this unweighted appropriation does not represent the exact population. Therefore, the region with a small population is oversampled to overcome these issues and is, for this reason, the weighted sample used in this study (NMCP and ICF, 2018; Gaston and Ramroop, 2020).

### 2.4 Blood collection and laboratory method

Children aged 6–59 months were tested for both anaemia and malaria with the parents' or guardians’ consent. Trained nurses were responsible for the testing and the children who tested positive were given medication on the spot, according to the national rules.

#### 2.4.1 Anaemia testing

Blood samples were collected from each child aged 6–59 months using a spring-loaded, sterile lancet to make a finger- or heel-prick. The drop of blood was collected in a microcuvette, and the Haemoglobin level analysed using a portable HemoCue analyzer. The results were available within 10 min and were given to the child's parent or guardian verbally and in writing (NMCP and ICF, 2018). Parents were encouraged to take the children with haemoglobin level less than 8 g/dl to the nearest health care facility for the follow-up. The parents were given a referral letter with the haemoglobin examination to show the staff at the health care office. The results from the anaemia test were captured on the Biomarker Questionnaire and on the handout left in the household that contained information on the causes and prevention of anaemia (NMCP and ICF, 2018).

#### 2.4.2 Malaria testing

The blood sample was collected from children's finger- or heel-prick using the SD Biolne Malaria Ag P.f/P, a rapid diagnostic test (RDT). Malaria testing can be done using microscopy or rapid diagnostic test; however, in this study the RDT was considered.

Microscopic diagnosis is helpful in testing malaria, although it does have some limitations; these include inadequately trained microscopists, lack of quality control, the chance of misdiagnosis because of low parasitemia or blended diseases, and in some cases it is hard to determine the types of plasmodium. Moreover, microscopy services are not accessible, for example, in a remote area or after standard laboratory hours (Ohrt et al., 2002; Wongsrichanalai et al., 2007; Gaston and Ramroop, 2020). Thus, for that reason, the RDT is used and is relevant in the detection of the histidine-rich protein II (HRP II). Furthermore, the RDT detects an antigen of Plasmodium falciparum and common Plasmodium lactate dehydrogenase (PLDH) of Plasmodium species in human blood (NMCP and ICF, 2018). The diagnostic test incorporates an expendable example tool that arrives in a standard bundle. A tiny volume of blood is captured on the applicator and placed in the well of the testing device. All field laboratory experts were upskilled to use the RDT in the field as per the producer's directions. The RDT results were accessible quickly and recorded as either positive or negative, with blackout test lines viewed positive. Likewise, with the anaemia testing, malaria RDT results were given to the child's parent or guardian in oral and composed structure and were recorded on the Biomarker Questionnaire. Moreover, children who tested positive for malaria were provided with a full course of medication as indicated by the standard system for uncomplicated malaria treatment in Malawi (NMCP and ICF, 2018).

## 3 Data analysis

### 3.1 Dependent variable

The present study considered two response variables or dependent variables. The first one was anaemia status for children 6–59 months of age. The anaemia status in children is determined based on the hemoglobin concentration level in the blood measured in grams per deciliter (g/dl). When the hemoglobin concentration level adjusted for altitude is less than 11 g/dl, the child is reviewed as anaemic, otherwise not anaemic (WHO, 2015; NMCP and ICF, 2018). The second one is malaria status using RDT to check if the child has malaria (positive) or not (negative).

### 3.2 Independent variables

The exploratory covariate or independent variables used in this study were also used in previous literature and involved a number of socio-economic, demographic, and geographic factors (Bennett et al., 2013; Alegana et al., 2014; Buchwald et al., 2016; Zgambo et al., 2017). The present study used independent variables assumed to be linked with malaria and (or) anaemia such as the child's age in months; the sex of the child; the type of residence; region of the dwelling; wealth quantile; mother's highest education level; source of drinking water; type of toilet facility; household sharing the toilet; household using electricity or not; children under 5 slept under a mosquito bed net the night before the survey; the main material of wall, floors; roofs of the rooms and residence altitude. In Malawi the studies which modelled anaemia and malaria also used the same independent variables found in the present study (Kabaghe et al., 2017; McGann et al., 2018; Yimgang et al., 2021). However, these studies did not include some variables assumed to be associated with anaemia and (or) malaria (Gaston and Ramroop, 2020). The independent variables which were not included in their studies are source of drinking water; type of toilet facility; household sharing the toilet; household using electricity or not; children under 5 slept under a mosquito bed net the night before the survey; the main material of wall, floors; roofs of the rooms and residence altitude.

## 4 Statistical analysis

The present study used Statistical Package for the Social Sciences (SPSS) to clean the data. In addition, the analysis of bivariate method was done in SPSS, with the application of cross-tabulation techniques. Pearson's chi-square test and p-values were used to investigate whether the independent variables were associated with each one of the responses variables or not. To summarize the data, the frequencies and percentages were used, and p-values to check the relationship between exploratory variables and response variables. Any exploratory variables with a p-value of less than 0.2 were included in the multivariate generalized linear mixed model (GLMM) (El Kishawi et al., 2015; Gaston et al., 2018). Then any exploratory variable found to have a statistically significant association with response variables at p-value less than 0.05 in multivariate was reported. The analysis of multivariate used SAS 9.4 PROC GLIMMIX procedure. The procedure enabled us to jointly model two outcomes (response) variables with similar distributions, link functions, or different link functions. However, this study assessed a similar distribution and link functions for the two outcome variables. Furthermore, numerous covariance structures were considered based on lowest value of Akaike information criteria (AIC) and Unstructured (UN) were found to be suitable to our analysis. In addition, we checked the possible interaction and none was statistically significant (Molenberghs and Verbeke, 2005; Habyarimana et al., 2016).

### 4.1 Model formulation

The study considered two response variables, which were malaria and the anaemic status of a child. Suppose the response variable $y_{i1}$ to be malaria RDT status, where one (1) is assigned as positive status and zero (0) negative status. The second response variable as $y_{i2}$ to be anaemia status, where one (1) is assigned as an anaemic child and zero (0) non-anaemic. When the distinguished results emerge from a bivariate Bernoulli distribution, with $p_{i1}$ as the likelihood of malaria occurring in a child *i* and $p_{i2}$ as the probability of anaemia occurring in a child *i.* In this way, the binary generalized linear model can be written as:

(1) $$g_{1}\left(\mu_{i1}\right)=X_{i1}\beta_{1}+Z_{i1}v_{1}$$

(2) $$g_{2}\left(\mu_{i1}\right)=X_{i2}\beta_{2}+Z_{i2}\;v_{2}$$

Where $\beta_{1}$ and $\beta_{2}$ are assumed to be the vectors of fixed effects, $v_{1}$ and $v_{2}$ are the vectors of the random effects, while$\;X_{i1}$,$\;X_{i2},\;Z_{i1}$ and $Z_{i2}$ are the designed matrices for fixed effects and random effects respectively. Thus, equation of the variance-covariance matrices model is shown as follows:

(3) $$v=\left(\begin{array}{l} v_{1}\\ v_{2} \end{array}\right)∼i.i.d.MVN\left(0,\sum \right)\;=MVN\left(\left[\begin{array}{l} 0\\ 0 \end{array}\right],\left[\begin{array}{ll} \sum_{11} & \sum_{12}\\ \sum_{21} & \sum_{22} \end{array}\right]\right)$$

where the $\sum_{11}$ and$\;\sum_{22}$, are the variance components of malaria of children under five years and anaemia respectively, while $\sum_{12}$ and $\sum_{21}$ are the correlation components between malaria and anaemia are the same. When the correlation components from Eq. (3), $\sum_{12}$ = $\sum_{21}$ = 0, the multivariate joint model under generalized linear mixed model becomes a separate model (Molenberghs and Verbeke, 2005; Habyarimana et al., 2016).

### 4.2 Results and interpretation

#### 4.2.1 Univariate results

The results from Table 1 and Table 2 indicated the frequency distribution and percentages of childhood anaemia and malaria respectively with the associated factors. Cross-tabulation techniques were used to analyse the data and summarise the results in both Tables. Pearson's chi-square test and p-values were used to investigate whether the independent variables were statistically significant associated with each one of the responses variables or not. The results from Table 1 indicated that all independent variables were significantly associated with childhood anaemia with the p-value less than 0.05, except for the sex of the child, and households who share a toilet. Table 2 also showed that all independent variables were significantly associated with childhood malaria with the p-value less than 0.05, except for the sex of the child. The prevalence of anaemia and malaria from this study was 56.9 % and 37.2% respectively.

**Table 1:** Childhood anaemia by categorical variable.

Variables	Category	Anaemia status		P-value
		Anaemic	Not anaemic	
Child's age in months	6–23 months	609 (78.6%)	166 (21.4%)	0.000
	24–31 months	466 (58.0%)	337 (42.0%)	
	32–59 months	474 (51.0%)	455 (49.0%)	
Sex of the child	Male	771 (61.0%)	492 (39.0%)	0.432
	Female	779 (62.6%)	466 (37.4%)	
Sleep under mosquito bed net	All children	956 (58.0%)	691 (42.0%)	0.002
	Some	136 (52.7%)	122 (47.3%)	
	None	458 (55.9%)	361 (41.1%)	
Region	North	145 (53.5%)	126 (46.5%)	0.004
	Central	686 (64.4%)	379 (35.6%)	
	South	718 (61.3%)	453 (38.7%)	
Place of residence	Rural	1366(63.0%)	801 (37.0%)	0.001
	Urban	184 (54.0%)	157 (46.0%)	
Wealth index	Poorer	793 (67.7%)	379 (32.3%)	0.000
	Middle	284 (59.2%)	196 (40.8%)	
	Richer	473 (55.2%)	384 (44.8%)	
Mother's education level	No education	226 (68.9%)	102 (31.1%)	0.000
	Primary	1125(55.8%)	891 (44.2%)	
	Post-primary	198(52.2%)	181 (47.8%)	
Altitude	0–500 m	240 (72.9%)	89 (27.1%)	0.000
	501–1000 m	604 (60.0%)	402 (40.0%)	
	>1000 m	706 (60.2%)	467 (39.8%)	
Toilet facility	Toilet with flush	198 (51.3%)	188 (48.7%)	0.000
	Pit latrine	1235(63.3%)	717 (36.7%)	
	No facility	117 (68.8%)	53 (31.2%)	
Type of drinking water	Tap water	339 (53.6%)	294 (46.4%)	0.024
	Protected water	290 (54.3%)	244 (45.7%)	
	Unprotected	920 (59.1%)	636 (40.9%)	
Main roof material	Thatch/Palm leaf	920 (61.4%)	579 (38.6%)	0.000
	Corrugated metal	557 (50.7%)	542 (49.3%)	
	Stick & mud	73 (57.5%)	54 (42.5%)	
Main wall material	Wood/Mud	475 (61.9%)	292 (38.1%)	0.001
	bricks	624 (53.6%)	540 (46.4%)	
	Cement/Block	451 (56.9%)	342 (43.1%)	
Main floor material	Earth/Sand	1143(59.2%)	789 (40.8%)	0.000
	Mud block/Wood	110 (58.5%)	78 (41.5%)	
	Cement/Block	297 (49.2%)	307 (50.8%)	
Electricity	Yes	122 (42.5%)	456 (44.6%)	0.000
	No	1427(58.6%)	654 (42.9%)	
Household share toilet	Yes	566 (55.4%)	456 (44.6%)	0.380
	No	872 (57.1%)	654 (42.9%)

**Table 2:** Childhood malaria by categorical variable.

Variables	Category	Malaria status		P-value
		Positive	Negative	
Child's age in months	6–23 months	200(25.6%)	581(74.4%)	0.000
	24–31 months	411(40.6%)	602(59.4%)	
	32–59 months	403(43.4%)	526(56.6%)	
Sex of the child	Male	463(36.5%)	804(63.5%)	0.716
	Female	447(35.8%)	800(64.2%)	
Sleep under mosquito bed net	All children	553(33.6%)	1093(66.4%)	0.000
	Some	88 (34.1%)	170 (65.9%)	
	None	372(45.4%)	447 (54.6%)	
Region	North	62 (20.8%)	236 (79.2%)	0.000
	Central	452(39.8%)	683 (60.2%)	
	South	500(38.7%)	791 (61.3%)	
Place of residence	Rural	987 (41.9%)	1369(58.1%)	0.000
	Urban	27 (7.3%)	341 (92.7%)	
Wealth index	Poorer	611(48.0%)	662(52.0%)	0.000
	Middle	204(39.6%)	311(60.4%)	
	Richer	199(21.3%)	737(78.7%)	
Mother's education level	No education	152(46.3%)	176(53.7%)	0.000
	Primary	799(39.6%)	1218(60.4%)	
	Post-primary	63 (16.6%)	216(83.4%)	
Altitude	0–500 m	119(34.3%)	228 (65.7%)	0.000
	501–1000 m	492(43.8%)	631 (56.2%)	
	>1000 m	403(32.2%)	850 (67.8%)	
Toilet facility	Toilet with flush	88 (21.1%)	330(78.9%)	0.000
	Pit latrine	832(39.3%)	1287(60.7%)	
	No facility	95 (50.8%)	92 (49.2%)	
Type of drinking water	Tap water	164(25.9%)	470 (74.1%)	0.000
	Protected water	122(31.9%)	260 (68.1%)	
	Unprotected	728(42.6%)	980 (57.4%)	
Main roof material	Thatch/Palm leaf	686(45.8%)	812 (54.2%)	0.000
	Corrugated metal	283(25.8%)	816 (74.2%)	
	Stick & Mud	45 (35.7%)	81 (64.3%)	
Main wall material	Wood/Mud	331(43.2%)	436 (56.8%)	0.000
	bricks	467(40.1%)	697 (59.9%)	
	Cement/Block	216(27.2%)	577 (72.8%)	
Main floor material	Earth/Sand	840(43.5%)	1092(56.5%)	0.000
	Mud block/Wood	74 (39.4%)	114 (60.6%)	
	Cement/Block	100(16.6%)	504 (83.4%)	
Electricity	Yes	21 (7.3%)	265 (92.7%)	0.000
	No	992(40.7%)	1445(59.3%)	
Household share toilet	Yes	329(32.2%)	692 (67.8%)	0.000
	No	600(39.3%)	927(60.7%)	

The results from Table 1 revealed that the prevalence of anaemia was higher in children from mothers with no education (68.9%) and lower with mothers with primary (55.8%) and post-primary (52.2%) respectively. The same results indicated a decrease in the prevalence of anaemia in children from wealthier classes (55.2%), and increase in middle (59.2%), and poorer classes (67.7%). The prevalence of anaemia in children from a rural area (63.0%) was higher compared to those from an urban area (54.0%). The results also indicated a decrease in prevalence of anaemia as the age for a child increased. The prevalence of anaemia was 78.6%, 58.0%, and 51.0% among children aged between 6-23, 24–41, and 42–59 months respectively.

The results from Table 2 revealed that the prevalence of malaria was higher in children from mothers with no education (46.3%), and reduced by mothers with primary (39.6%) and post-primary education (16.6%) respectively. The same results indicated that the prevalence of malaria was lower in children from the wealthier (21.3%), and increased to middle (39.6%) and poorer (48.0%) classes respectively. The prevalence of malaria in children from rural area was higher (41.9%); while for those from the urban area it was lower (7.3%). Table 2 also showed that the prevalence of malaria increased as the child's age increased, which was 25.6%, 40.6%, and 43.4% among children aged between 6-23, 24–41, and 42–59 months respectively.

#### 4.2.2 Multivariate results

The analysis of multivariate used SAS 9.4 PROC GLIMMIX procedure to assess the correlation between anaemia and malaria and any other covariates factors which might be associated with the two diseases. In addition, we checked all possible interaction effects between the exploratory variables and none was statistically significant, hence these were not included in the results.

The results from Table 3 indicated the parameter estimates, odds ratio (OR), 95% confidence intervals (CI) and p-values. The study reported only the exploratory variables with statistically significant impact on malaria and anaemia (p < 0.05). The variables with a significant effect on both malaria and anaemia were the child's age, mother's education level, availability of electricity, toilet facilities, and children under five who slept under a mosquito bed net the night before the survey. The residence of household and altitude of residence had only a statistically significant effect on malaria.

**Table 3:** Parameter estimates for a joint marginal model for malaria and anaemia.

Indicator variables	Malaria				Anemia			
	Estimate	OR^a^	95% CI^b^ Upper; Lower	P-value	Estimate	OR	95% CI Upper; Lower	P-value
Child's age (Ref: 42–59)	-	-	-	-	-	-	-	-
6–23 months	-1.003	0.367	0.274; 0.490	0.000	1.456	4.289	3.418; 5.382	0.000
24–41 months	-0.069	0.933	0.728; 1.197	0.589	-0.008	0.992	0.815; 1.207	0.939
Residence (Ref: Urban)	-	-	-	-	-	-	-	-
Rural	1.284	-3.611	2.111; 6.178	0.000	-0.149	0.856	0.649; 1.145	0.303
Altitude (Ref: 501–1000 m)	-	-	-	-	-	-	-	-
>1000 m	-0.866	0.421	0.244; 0.725	0.002	-0.090	0.914	0.698; 1.197	0.512
0–500 m	-0.497	0.608	0.295; 1.256	0.179	0.280	1.323	0.919; 1.906	0.131
Education (Ref: No education)	-	-	-	-	-	-	-	-
Primary	-0.062	0.940	0.649; 1.361	0.743	-0.342	0.710	0.506; 0.997	0.048
Post-primary	-0.684	0.505	0.305; 0.835	0.008	-0.325	0.723	0.486; 1.075	0.109
Electricity (Ref: Yes)	-	-	-	-	-	-	-	-
No	0.831	2.296	1.415; 3.745	0.001	0.277	1.279	1.005; 1.732	0.046
Toilet facilities (Ref: No facility)	-	-	-	-	-	-	-	-
Pit latrine	-0.470	0.625	0.401; 0.975	0.038	-0.222	0.801	0.537; 5.930	0.277
Toilet with flush	-0.755	0.470	0.271; 0.815	0.007	-0.544	0.580	0.369; 0.913	0.019
Child sleeping under net (Ref: All children)	-	-	-	-	-	-	-	-
Some children	0.024	1.024	0.691; 1.516	0.906	0.364	1.439	1.064; 1.946	0.018
No children	0.461	1.586	1.045; 2.406	0.030	0.055	1.057	0.763; 1.462	0.742

a OR: odds ratio.

b 95% CI: confidence interval.

The results from Table 3 indicated that children aged 6–23 months were 0.367 (OR: 0.367, 95% CI: 0.274; 0.490) times less likely to test positive for malaria using an RDT when compared with those in the reference group (42–59 months). In contrast, the children aged 6–23 months were 4.289 (OR: 4.289, 95% CI: 3.418; 5.382) times more likely to have anaemia compared with those in the age group 42–59 months.

The same results showed that children from mothers with post-primary levels were 0.505 (OR: 0.505, 95% CI: 0.305; 0.835) times less likely to have malaria compared to those from the mother with no education. The children from mothers with primary school were 0.710 (OR: 0.710, 95% CI: 0.506; 0.997) times less likely to have anaemia compared with those in the reference category group. The results revealed that the odds of testing positive malaria in an RDT for children from households with no access to electricity were 2.296 (OR: 2.296, 95% CI: 1.415; 3.745) times more likely than those from households with access to electricity. The same results indicated that children from households with no access to electricity were 1.279 (OR: 1.279, 95% CI: 1.005; 1.732) times more likely to have anaemia compared to those from households with access to electricity.

The study also revealed that children from a household with pit latrines were 0.625 (OR: 0.625, 95% CI: 0.401; 0.975) times less likely to test positive for malaria than those with no toilet facilities, while those from households with flush toilet were 0.470 (OR: 0.470, 95% CI: 0.271; 0.815) times less likely to have malaria compare to those with no toilet facilities. Furthermore, children from households with flush toilets were 0.580 (OR: 0.580, 95% CI: 0.369; 0.913) times less likely to have anaemia, compared to those with no toilet facilities.

The results indicated that the odds of testing positive malaria in an RDT was 1.586 (OR: 1.586, 95% CI: 1.045; 2.406) times more likely in households with no children slept under a mosquito bed net the night before the survey, compared to households with all children slept under a mosquito bed net.

However, the odds of having anaemia in some children who slept under a mosquito bed net the night before the survey, were 1.439 (OR: 1.439, 95% CI: 1.064; 1.946) times more likely than all children who slept under a mosquito bed net.

Table 3 showed that children living in rural areas were 3.611 (OR: 3.611, 95% CI: 2.111; 6.178) times more likely to test positive for malaria compared to those living in urban areas. The same results indicated that children who live in a residence with an altitude of more than 1000 m were 0.421 (OR: 0.421, 95% CI: 0.244; 0.725) times less likely to have malaria than those living in residence with an altitude between 501 and 1000 m.

However, the children living in rural and altitude residence areas were not statistically significant to anaemia, hence we did not interpret the results.

Table 4 shows the variance components and covariance between anaemia and malaria. The coefficient estimate of 0.700 indicates the relationship between anaemia and malaria. This means that there is a statistically significant positive correlation between malaria and anaemia.

**Table 4:** Variance components.

Variables	Estimates	Odds Ratio (OR)	95% CI (Upper, Lower)	P-value
Variance (Malaria)	1.014	2.757	0.610; 1.418	0.000
Variance (Anaemia)	0.118	1.125	0.020; 0.216	0.009
Correlation between Anaemia and Malaria	0.700	2.014	0.333; 1.067	0.000

The overall fitted model was highly significant as the coefficient of covariance parameter indicated the p-value = 0.000. Hence, including the random cluster effect in the model was necessary (Molenberghs and Verbeke, 2005; Zhang and Lin, 2008).

The pseudo-likelihood test rejected the null hypothesis of zero correlation with Pearson chi-squared test = 4591.22 and the ratio of Pearson and degree of freedom of 0.91 with p-value = 0.000. This revealed that there is a good variability in the dataset and there was no residual over dispersion (Molenberghs and Verbeke, 2005). Furthermore, this indicated that the association between the prevalence of malaria and anaemia was highly significant. In addition, the odds ratio (2.014) also confirmed that anaemia and malaria are highly associated. The results from the present study are in line with the study by Seyoum (2018).

## 5 Discussion

The present study utilized the joint model for a multivariate generalized linear mixed model (GLMM) to simultaneously model the association between malaria and anaemia and identify factors associated with the two diseases. The study indicated that anaemia and malaria are highly associated. This means that malaria and anaemia move in the same direction, where any increase of malaria in children, will also result in an increase of anaemia. This finding is consistent with existing literature (Noland et al., 2012; Zgambo et al., 2017). The same change can be in a negative direction; when malaria reduces in children, so does anaemia. Therefore, the change between both malaria and anaemia can be interpreted to mean that controlling malaria can result in effectively reducing anaemia (Reithinger et al., 2013; Hershey et al., 2017; Yimgang et al., 2021). In addition, controlling anaemia in the area of high prevalence of malaria can result in reduction of deaths related to malaria (Korenromp et al., 2004; Seyoum, 2018).

The findings from this study revealed that children from mothers with primary and post-primary education level were less likely to have both malaria and anaemia compared to those from mothers with no education. This shows that the risk of having malaria or anaemia reduces as the education levels of their mothers increases. The findings from this study are aligned with the studies by Adebayo et al. (2016), Seyoum (2018), and Gaston and Ramroop (2020).

The results indicated that children from households with no access to electricity have more risk of having malaria and anaemia. Furthermore, households with no toilet facilities are more likely to increase the rates of positive malaria RDT and anaemia. Access to electricity and toilet facilities are indicators of socio-economics. Therefore, households with access to socio-economics can easily access healthcare, are able to eat healthy food and can easily afford the treatment (Ayele et al., 2014b; Gaston and Ramroop, 2020).

The findings from the present study also indicated that children who did not sleep under a mosquito bed net the night before the survey had more risk of having malaria and anaemia. This might be due to the children who sleep under mosquito bed nets being more protected from being bitten by *Anopheles* mosquitoes which is the cause of malaria (Gaston and Ramroop, 2020). The same results were found in previous studies, such as those by Ayele et al. (2014) and Zgambo et al. (2017).

The research showed that the children living in high altitude residence are less likely to have malaria. This is due to the markedly higher temperature at lower altitude residence area, where the mosquitoes develop in hotter areas (Chirombo et al., 2014; Teh et al., 2018; Gaston and Ramroop, 2020). The results also revealed that children from rural area are more likely to test positive for malaria in an RDT. The findings from this study are in line with the studies by Adebayo et al. (2016), and Gaston and Ramroop (2020).

The findings from this study revealed that the probability of a positive malaria increased as the child's age increased. The children aged 6–23 months were less likely to test positive for malaria. These results are in contrast with the results found in the study by Seyoum (2018). However, the same results are in line with the studies by Zgambo et al. (2017), Gaston and Ramroop (2020), and Yimgang et al. (2021).

In contrast, the probability of having anaemia reduced as the child's age increased. Children aged 6–23 months were more likely to have anaemia. This might be due to the fact that anaemia is also associated with other factors such as nutritional deficiencies, diseases infections such as HIV, intestinal worms, intake of iron, folate, vitamin B12, and other parasitic infections (Ayoya et al., 2013; Gaston et al., 2018). Furthermore, the immune system of young children is not strong enough to fight against different diseases but becomes stronger as they grow older. Thus, for this self-same reason young children are highly at risk of getting anaemia, and this reduces as they grow older. The results from this study are consistent with the studies by Hershey et al. (2017), Kuziga et al. (2017), Gaston et al. (2018), and Yimgang et al. (2021).

## 6 Conclusion

The main objective of this cross-sectional study was to examine the association between malaria and anaemia using a multivariate joint model under the GLMM in children 6–59 months of age in Malawi. The current scientific setting also checked other factors which might be associated with both malaria and anaemia. Finally, we examined all possible interaction effects between the exploratory variables and these were not included in the results since none was statistically significant.

The findings from this study indicate that there is an association between anaemia and malaria and any change in one disease has a similar effect on the other disease. This means that as malaria increases so does anaemia and vice versa. Therefore, the study suggests that any policy change to malaria will impact on anaemia. Furthermore, the two diseases are associated with socio-economic, demographic, and geographical factors, which makes these diseases a persistent and a current problem.

Based on the findings from this study, there is a need for educating the population, particularly those from rural areas, on how to prevent malaria and anaemia in children under five years of age. The policy makers and Malawian government should focus on improving the toilet facilities, access to electricity and providing more mosquito bed nets, mostly for the individuals who live in rural area and at low altitude. In addition, education of the mothers should be prioritized so that they are able to treat and take care of their children, especially those in the age group 6–23 months, as they are more vulnerable. Understanding the relationship between anaemia and malaria together with other factors associated with the two diseases can provide useful insights to the government and policy makers in planning, controlling and the elimination of both diseases. In addition, the statistical model used in this study will help other researchers to compare findings and referencing. Future research could make use of spatial joint models for malaria and anaemia simultaneously in order to investigate the correlation between the two diseases by geographical location.

## 7 Limitation

The MMIS 2017, under household member dataset, did not provide the nutritional status of the child, cough and diarrhea effects, which might be associated with anaemia. It would be helpful to use a dataset that included those factors. The second limitation is that the dataset was cross-sectional. It would have been ideal to have a longitudinal data set to study the change in factors and prevalence over time.

## 8 Recommendations

In order to develop effective intervention strategies to help reduce or alleviate anaemia and malaria in children aged 6–59 months, it is recommended that the policy makers and government make a concerted effort in educating the mothers on malaria and anaemia, and improving health care, toilet facilities and mosquito bed nets, especially for individuals from rural areas. This can be done through various platforms such as social media, television, radio, roadshows and even rural based workshops.

## Declarations

### Author contribution statement

Rugiranka Tony Gaston: Conceived and designed the experiments; Performed the experiments; Analyzed and interpreted the data; Contributed reagents, materials, analysis tools or data; Wrote the paper.

Faustin Habyarimana and Shaun Ramroop: Conceived and designed the experiments; Contributed reagents, materials, analysis tools or data.

### Funding statement

This work was supported by the Health Economics and HIV/AIDS Research Division (HEARD).

### Data availability statement

The data that has been used is confidential.

### Declaration of interests statement

The authors declare no conflict of interest.

### Additional information

No additional information is available for this paper.
